# Dietary administration of probiotics modulates non-specific immunity and gut microbiota of Nile tilapia (*Oreochromis niloticus*) cultured in low input ponds

**DOI:** 10.1080/23144599.2019.1624299

**Published:** 2019-06-30

**Authors:** Mary A. Opiyo, James Jumbe, Charles C. Ngugi, Harrison Charo-Karisa

**Affiliations:** a Department of Zoological Sciences, Kenyatta University, Nairobi, Kenya; b Kenya Marine and Fisheries Research Institute, National Aquaculture Research Development and Training Center, Sagana, Kenya; c Department of Natural Resources, Karatina University, Karatina, Kenya; d WorldFish, Sustainable Aquaculture Program Cairo Office, Maadi, Cairo, Egypt

**Keywords:** Bacillus subtilis, immunity, Nile tilapia, probiotics, Saccharomyces cerevisiae

## Abstract

Poor culture conditions in low input ponds make fish highly susceptible to infectious pathogens which lead to diseases and mortalities yet the effects of probiotics on immunity, gut microbiota and microbiological quality of fish in low input ponds are unknown. Nile tilapia, *Oreochromis niloticus* fingerlings (40 g) were randomly stocked at 50 fish m^−3^ in 1.25 m^3^ cages in low input ponds. The fish were fed on diets supplemented with either *Saccharomyces cerevisiae* (1 × 10^10^ CFU g^−1^) or *Bacillus subtilis* (1 × 10^9^ CFU g^−1^) at six levels: Diet 0 (No probiotic); *S. cerevisiae* at 2 g kg^−1^ (Diet 1); 4 g kg^−1^ (Diet 2) and 6 g kg^−1^ (Diet 3) and *B. subtilis* at 5 g kg^−1^ (Diet 4); 10 g kg^−1^ (Diet 5) and 15 g kg^−1^ (Diet 6) for 180 days. Results indicate that hemato-immunological parameters (hemoglobin (Hb), red blood cells (RBC), white blood cells (WBC) serum protein, albumin, globulin and lysozyme activity) were significantly higher in fish fed on probiotic treated diets compared to the control (*P* < 0.05). On the contrary, fish fed on Diet 6 presented significantly similar Hb and globulin values compared to the control (P > 0.05). Additionally, fish fed on probiotic treated diets retained the probiotics in their guts and lower microbial load was realized in their muscle (*P* < 0.05). In conclusion, *B. subtilis* and *S. cerevisiae* supplementation in diets of Nile tilapia reared in low input ponds improves immunity, manipulates gut microbiota and enhances fish flesh quality.

## 1. Introduction

In most parts of Asia and Africa, farming of Nile tilapia (*Oreochromis niloticus*) mainly occurs in low input ponds where supplementary feeding is done alongside pond fertilization to reduce the cost of feeds [1,2]. Although low input systems are well established in many parts of the world [3–5], previous studies have indicated that only 5–15% of the nutrients input in fertilized pond systems are converted to harvestable products leading to excess nutrients in form of Nitrogen (N), Phosphorus (P) and organic matter [6]. These excess nutrients usually result from feed remnants, faeces and excreta and often lead to eutrophication, water quality problems, physiological stress, high susceptibility to pathogens and mortality of the cultured fish [7–10]. Additionally, pathogenic bacteria can also be introduced into the culture environment via organic manure, through incoming water and feeds exposing fish to infections [11].

Cases of increase in pathogenic bacteria populations e.g. *Vibrio* spp., *Salmonella* spp. and *Aeromonas* spp. which pose a health risk to the cultured fish, rendering them immune-compromised, have been reported in fertilized ponds [12,13]. Moreover, fish has an intimate interaction with the culture environment which has both pathogenic and saprophytic microorganisms [10,14]. Apart from the skin and gills, the gastrointestinal tract of fish is a key area of interaction with pathogens present in culture water. Therefore, enhancement of the immune system of cultured fish and establishment of normal gut microbiota is vital, as it affects a wide range of biological processes including development and assembly of gut-associated lymphoid tissue (GALT) and ability to fight infections [15,16]. The non-specific (innate) immune defense system of fish is considered as the first line of defense against pathogens [17,18].

In the past decade, interest in the use of probiotics for modulation of the non-specific immune system of fish against diseases has been increasing [19–24]. Dietary administration of bakers’ yeast, *Saccharomyces cerevisiae* and *Bacillus subtilis* have been used to improve immune response in different species of fish [21,23–31]. However, despite these studies, it is still worth noting that the application of probiotics in low input ponds remain unknown yet their effects could be more striking in poor conditions exhibited in low input ponds [32]. This study investigated the effects of commonly used probiotics on non-specific immunity, gut microbiota and microbiological quality of Nile tilapia reared in low input ponds.

## 2. Materials and methods

### 2.1. Experimental design

This study was conducted at Kenya Marine and Fisheries Research Institute, Sagana (altitude 1230 m, latitude 0°39´S and longitude 37°12´E) located in Sagana town in Kenya. The study was conducted for a period of 180 days (November 2016–April 2017). Net cages measuring 1.25 m^3^ (1.0 × 1.0 × 1.25 m); mesh size 1.80 cm were placed in four earthen ponds each measuring 150 m^2^; with each pond holding seven cages. Each cage was floated by wooden bars 30 cm above the pond bottom and 25 cm above the water surface to prevent relocation. The cages were placed 3 m from each other and approximately 3 m away from either sides of the ponds. Each cage was fitted with a 30 cm diameter feeding-ring suspended at the midpoint of each cage to prevent feed spillage. Based on the experimental design, each cage represented one dietary treatment (Table 1); with all the seven treatments represented in each pond.

**Table 1.** Ingredient composition and chemical proximate composition of the experimental diets.

Ingredients (g kg^−1^)	Experimental Diets						
	Diet 0 (control)	Diet 1	Diet 2	Diet 3	Diet 4	Diet 5	Diet 6
Fish meal	190	190	190	190	190	190	190
Wheat bran	390	390	390	390	390	390	390
Wheat pollard	160	160	160	160	160	160	160
Maize germ	120	120	120	120	120	120	120
Cotton seed cake	120	120	120	120	120	120	120
Soybean oil	20	20	20	20	20	20	20
*S. cerevisiae*	0	2	4	6	0	0	0
*B. subtilis*	0	0	0	0	5	10	15
Chemical analysis (% of dry matter)							
Dry matter	88.9	89.2	88.3	87.9	88.4	87.6	88.7
Crude protein	29.4	29.9	30.2	29.7	29.3	29.9	29.4
Crude lipids	3.4	4.1	3.4	3.1	3.8	3.5	4.1
Crude fiber	5.7	6	6.2	4.7	5.1	5.8	6.1
Moisture	8.2	9.2	9.2	9.1	8.4	8.6	8.7
Ash	8.9	8.9	7.5	9.2	10.4	10.9	11.3

^a^*Saccharomyces cerevisiae*: – Diet 1 (2 g kg^−1^); Diet 2 (4 g kg^−1^) and Diet 3 (6 g kg^−1^)

^b^*Bacillus subtilis: –* Diet 4 (5 g kg^−1^); Diet 5 (10 g kg^−1^) and Diet 6 (15 g kg^−1^).

### 2.2. Experimental diets preparation

Dry feed ingredients were used to formulate the control diet. Different experimental diets were prepared by supplementing the control diet with dietary commercial probiotic *Saccharomyces cerevisiae*^a^ (1 × 10^10^ CFU g^−1^) FURAHA*®* (Agro-Chemical and Food Company Limited, Kenya) at 3 concentrations of 2 g kg^−1^ (Diet 1); 4 g kg^−1^ (Diet 2) and 6 g kg^−1^(Diet 3); and *Bacillus subtilis*^b^ (1 × 10^9^ CFU g^−1^) ULTRALACT*®* (Gee Dee Enterprises, India) at 3 concentrations of 5 g kg^−1^ (Diet 4); 10 g kg^−1^ (Diet 5) and 15 g kg^−1^ (Diet 6) according to Abdel-Tawwab et al. [27] and Hai [22]. The control diet (Diet 0), was not supplemented with any probiotic. All ingredients were thoroughly mixed with soybean oil at 20 g kg^−1^ of the feed and pelleted using an electric meat mincer (2 and 3 mm die). All male, monosex *O. niloticus* fingerlings of an average weight of 40 g were acclimatized on a control diet for 10 days after which they were randomly assigned to the seven treatments in four replicates. The experimental fish were stocked in net cages installed in the ponds at 50 fish m^−3^ according to Yi et al [33]. and Chakraborty [34].

### 2.3. Pond fertilization and fish feeding

Ponds were dried and limed using agricultural lime (CaCO_3_) according to the pH of the pond as described by Pillai and Boyd [35]. Two weeks prior to stocking, the ponds were fertilized with dry chicken manure at 50 g m^−2^ of dry matter and thereafter, fertilization was done on a weekly basis to stimulate natural productivity in low input ponds as recommended by Charo-Karisa [36]. The prepared diets were dried at room temperature, packed in plastic bags and refrigerated at 4°C to maintain the microbial viability before being fed to experimental fish [37]. New diets were prepared every two weeks to ensure high levels of probiotics remained in the diet during the experimental period. Fish were hand-fed twice daily at 1000 h and 1500 h at 3% of the total fish biomass.

### 2.4. Blood sample collection

At the end of the study, three fish from each replicate were anaesthetized with clove oil (20 mg L^−1^). Blood samples (1 ml from each fish) were drawn from the caudal vein of each fish using a sterile syringe, previously rinsed with 2.7% Ethylenediaminetetraacetic acid (EDTA) solution as an anticoagulant. The blood samples were used immediately for analysis of hemoglobin, red blood cells (RBC) and white blood cells (WBC). Extra blood (2 ml from each fish)was collected without anticoagulant and allowed to clot for 2 h in Eppendorf tubes and centrifuged at 3000 rpm using an Eppendorf centrifuge (Centrifuge 5415 R®) for 10 min. Blood serum was collected with a micropipette and stored at −20°C in Eppendorf tubes for analysis of serum total protein, albumin and lysozyme activity.

### 2.5. Hemato-immunological parameters analysis

Hemoglobin (Hb) was determined by a commercial hemoglobin kit (Marienfeld®, Germany) using Sahli’s/acid hematin method described by Wintrobe and Greer [38]. Red blood cells (RBC) and white blood cells (WBC) were counted after dilution with respective diluting fluids prepared according to Svobodová et al. [39]. Twenty microlitres of blood were mixed with 3980 µL of diluting fluid in a clean glass vial. The mixture was shaken well to suspend the cells uniformly in the solution. The red blood cell (RBC: 10^6^ mm^−3^) and white blood cells (WBC: 10^4^ mm^−3^) were counted using haemocytometer (Marienfeld®, Germany).

Serum lysozyme activity was determined using commercial fish lysozyme, enzyme-linked immunosorbent assay (ELISA) kits (Mybiosource®, USA). Optical densities (O.D) of the well plates were read in an ELISA plate reader (BioTek Powerwave ELx808 Microplate Reader/KC Junior software) at 450 nm. Serum total protein was determined by Bicinchoninic Acid (BCA) method using commercial total protein assay kit (Mybiosource®, USA). The samples were diluted 9 times with saline water before the assay. The absorbance of the standard and sample was measured against a blank in an ELISA plate reader (BioTek Powerwave ELx808® Microplate Reader/KC Junior software) at O.D of 630 nm. The absorbance readings were fitted in a normal curve and the value derived from the curve. Serum total protein was calculated using the following formulae:-

Serum total protein (µg dl^−1^) = (O.D sample) – (O.D blank)/O.D standard-O.D blank ×562 µg L^−1^× Dilution factor of the sample before testing.

Serum albumin was measured using commercial fish serum albumin ELISA kits (Mybiosource®, USA). The O.D of the well plates was read in an ELISA plate reader at 450 nm. Serum globulin was determined by subtracting the albumin values from the total serum protein. The albumin: globulin (A/G) ratio was calculated by dividing albumin by globulin values.

### 2.6. Fish muscle microbiological analysis and identification

Microbiological analysis of fish muscle was performed according to the standard procedures for enumeration of the respective group of microorganisms [40,41]. All equipment, chemicals and media were sterilized at 121ºC (15 lb pressure) for 15 minutes before use. Three whole fish from each treatment were rinsed with de-ionized water and the surface of the fish sterilized using 70% ethyl alcohol. Ten grams of muscle along with skin were taken randomly from different parts of the fish and were homogenized for 1 min with 90 ml of sterile saline (0.85% sodium chloride) solution in a stomacher – 400 lab blender. The homogenate was serially diluted to 10^−2^ and 10^−4^ for bacterial and yeast analysis respectively. Total plate count of aerobic bacteria was done by spread-plating 0.1 ml of the diluents in tryptone soy agar and incubated at 37°C for 16 h. The colony forming units (CFUs) were counted from each plate using a colony counter. Total coliforms were estimated by membrane filtration method where 1 ml of the homogenate was aseptically filtered through a membrane filter (Whatman filter pore diameter 0.45 µm) placed on Eosin Methylene Blue (EMB) agar and incubated at 37°C for 24 h. Typical *Escherichia coli* colonies were counted. Aliquots of the homogenate were inoculated into lactose broth in Durham tubes and incubated at 44.5°C for 24 h to selectively isolate faecal coliforms. Sub-culturing was done from tubes with gas in the Durham tubes on MacConkey sorbitol agar and incubated at 37°C for 48 h. Translucent to white colonies (*no sorbitol fermentation*) were purified for biochemical characterization.

*Salmonella* spp. were selectively isolated by inoculating aliquots of the homogenate in selenite enrichment broth and thereafter incubated at 37°C for 16 h. A loopful from each tube was sub-cultured on *Salmonella Shigella* agar (SS) and deoxycholate citrate agar (DCA). In cases where the colonies did not ferment lactose and/or produced hydrogen sulphide they were further purified for biochemical characterization. Pure culture isolates were identified based on colony morphology, gram stain and biochemical characterization according to Holt et al. [42]. These tests included triple sugar iron (TSI) test, sulphide production, motility, citrate utilization, urease test, methyl red and Voges Proskauer tests. Total yeast cells counts were done by spread plating 0.1 ml of the homogenate on Sabouraud’s agar and incubation was done at 25°C for 5 days. Microorganisms were identified up to the genus level according to MacFaddin [40]. Readings obtained with 30–300 colonies on the plate were used to calculate bacterial and yeast populations. Colony counts were log transformed and recorded as log CFU g ^−1^ of muscle [43].

### 2.7. Analysis and identification of gut microbiota

At the end of the feeding period, fish were starved for 24 h to allow gut evacuation and a random sample of 3 fish were taken from each treatment. Fish were sacrificed, dissected and longitudinally opened. The entire fish intestine was aseptically removed and homogenized in 90 ml, 0.85% sodium chloride solution in a stomacher – 400 lab blender. The final suspension was coarse-sieved using sterile nylon mesh (100 µm). Homogenates were serially diluted to 10^−4^ in 9 ml volumes of sterile 0.85% saline solution. Total plate count was done by spread plating 0.1 ml of each homogenate on tryptone soy agar (TSA) and incubation was done at 37°C for 16 h. Yeasts cells enumeration was done by spread-plating 0.1 ml of the homogenate on Sabouraud’s agar. The plates were incubated at 25°C for 5 days and yeast cells counted using a colony counter. Dominant colonies were purified and identified based on morphological characteristics and growth parameters using biochemical tests and standard techniques for isolating *Bacillus* spp. and yeast [40–42]. The bacterial and yeast cell counts were expressed as log CFU g^−1^ intestine.

### 2.8. Data analysis

All data were expressed as means ± standard error of the mean (SEM). Quantities of bacteria and yeast cells counts in the gut and muscle were log transformed before analysis. One-way ANOVA test was used to test significant differences among the groups at *P* ≤ 0.05. When overall differences were found, Tukey HSD test was used for pairwise comparisons between groups at *P* ≤ 0.05. All analyses were carried out using the Statistical Product and Service Solutions (SPSS version 20).

## 3. Results

### 3.1. Hemato-immunological parameters

Results of the hemato-immunological analysis are summarized in Table 2. Fish fed on diets containing probiotics had higher hemoglobin than the control group. However, the increase was not proportional to the probiotic inclusion level, with fish fed on Diet 5 having the highest hemoglobin. WBC increased with increase in probiotic treatment but reduced at the highest treatment. The highest value of WBC (60.83 × 10^4^ mm^−3^) was recorded in fish fed on Diet 2 with the lowest (41.40 × 10^4^ mm^−3^) in the control group. RBC and WBC counts were significantly higher (*P* < 0.05) in fish fed on Diet 2 and Diet 5 respectively. However; no significant differences was recorded for hemoglobin and RBC between fish fed on Diet 0 and Diet 6 (*P* > 0.05).

**Table 2.** Hemato-immunological parameters of *O. niloticus* fed on *S. cerevisiae* and *B. subtilis* treated diets in low input ponds.

	Diet						
Parameter	Diet 0	Diet 1	Diet 2	Diet 3	Diet 4	Diet 5	Diet 6
Hemoglobin (g dl^−1^)	4.46 ± 0.39^a^	6.66 ± 0.41^b^	7.28 ± 0.39^b^	6.76 ± 0.47 ^b^	5.14 ± 0.33^a^	7.71 ± 0.27^b^	5.10 ± 0.19^a^
RBC (10^6^ mm^−3^)	1.67 ± 0.17^a^	2.03 ± 0.18^a^	2.97 ± 0.19^ab^	2.46 ± 0.26^a^	2.25 ± 0.20^ac^	3.11 ± 0.17^d^	2.69 ± 0.15^a^
WBC (10^4^ mm^−3^)	41.40 ± 2.99^a^	51.04 ± 2.30^b^	60.83 ± 4.23^b^	51.58 ± 3.27^bc^	51.40 ± 1.65^b^	56.43 ± 1.61^b^	50.38 ± 1.88^b^
Total protein (µg dl^−1^)	4.11 ± 0.30^a^	5.07 ± 0.23^b^	5.12 ± 0.44^b^	5.30 ± 0.41^b^	4.85 ± 0.22^ab^	4.95 ± 0.19^ab^	5.22 ± 0.21^b^
Total albumin (µg dl^−1^)	1.55 ± 0.22^a^	1.79 ± 0.22^b^	1.86 ± 0.45^ab^	2.20 ± 0.28^b^	2.15 ± 0.19^b^	2.49 ± 0.21^b^	2.56 ± 0.15^b^
Globulin (µg dl^−1^)	2.34 ± 0.47^a^	3.21 ± 0.55^ab^	3.31 ± 0.34^b^	3.10 ± 0.55^b^	2.70 ± 0.31^c^	2.74 ± 0.30^c^	2.38 ± 0.31^a^
Albumin globulin (A/G) ratio	2.11 ± 1.00^a^	1.01 ± 0.47^ab^	0.57 ± 0.07^b^	1.26 ± 0.49^b^	0.97 ± 0.21^b^	1.09 ± 0.25^b^	1.68 ± 0.54^ab^
Lysozyme activity (U ml^−1^)	9.42 ± 1.51^a^	11.86 ± 1.46^b^	18.00 ± 3.11^c^	14.51 ± 1.99^b^	11.96 ± 0.79^b^	17.56 ± 5.46^c^	15.02 ± 1.51^b^

^a^Data represented as means ± SEM (n = 12). Mean values in the same row having different superscript letters (a, b and c) are significantly different at *P* ≤ 0.05.

Serum protein and albumin increased with increase in *S. cerevisiae and B. subtilis* dosage in the diets. Fish fed on Diet 6 exhibited significantly higher serum protein and albumin levels (*P* < 0.05) compared to the control. However, no significant differences were found in serum albumin (*P* > 0.05) in fish fed on Diet 4, 5 and 6. Serum globulin and lysozyme activity were significantly affected by different dosages of probiotics (*P* < 0.05) and increased with increase in probiotic dosage. Nevertheless, both serum globulin and lysozyme activity were significantly lower at the highest dosage of each probiotic (*P* < 0.05). The lowest globulin level was recorded in fish fed the control diet but was not significantly different from fish fed on Diet 6 (*P* > 0.05). Fish fed on Diet 1, 2 and 3 had significantly higher globulin levels (*P* < 0.05) compared to those fed on Diet 4, 5 and 6. Lysozyme activity ranged between 6.17 to 20.50 U ml^−1^ and was not significantly different in fish fed on Diet 1, 3, 4 and 6 (*P* > 0.05). The albumin/globulin ratio decreased significantly in all the fish fed probiotic treated diets compared to the control (*P* < 0.05).

### 3.2. Fish muscle microbiological content

Total plate count (TPC), *Escherichia coli*, faecal coliform, *Salmonella* spp. and total yeast cells counts in fish fed on probiotic treated diets are shown in Table 3. Probiotic treatments resulted in low microbial counts in the muscle of Nile tilapia. High total plate counts were found in fish fed on the control diet (2.27 × 10^−2^ log CFU g^−1^) while the lowest counts were recorded in fish fed on Diet 2 (1.44 × 10^−2^ log CFU g^−1^). *E. coli* counts were significantly higher in the control group compared to fish fed on probiotic treated diets (*P* < 0.05). Faecal coliform and *Salmonella* spp. were not detected (n.d) in fish fed on probiotic treated diets and were only detected in the control group with mean levels of 1.14 × 10^−2^ and 1.01 × 10^−2^ log CFU g^−1^ respectively. Probiotic treatment significantly affected the total yeast cells counts *(P *< 0.05) with higher values being recorded in the control group compared to other treatments. Fish fed on Diet 5 had significantly lower yeast cell counts than the control (*P* < 0.05). It is noted that the results of this study are lower than the limits of safety acceptance recommended for microbiological limits for fish [44].

**Table 3.** The microbial content of muscle of *O. niloticus* fed on *S. cerevisiae* and *B. subtilis* treated diets in low input ponds.

Parameter	Diet						
	Diet 0	Diet 1	Diet 2	Diet 3	Diet 4	Diet 5	Diet 6
Total plate count (log CFU g^−1^) (10^−2^)	2.27 ± 0.16^ac^	1.83 ± 0.22^bc^	1.44 ± 0.09 ^b^	1.61 ± 0.31^bc^	2.08 ± 0.01^bc^	1.49 ± 0.07^b^	2.00 ± 0.03^bc^
*E. Coli* (log CFU g^−1^) (10^−2^)	1.99 ± 0.05^a^	1.75 ± 0.04^bc^	1.48 ± 0.04^c^	1.70 ± 0.02^cd^	1.82 ± 0.04^ac^	1.59 ± 0.05^c^	1.80 ± 0.03^ac^
Feacal coliform (log CFU g^−1^) (10^−2^)	1.14 ± 0.03	n.d	n.d	n.d	n.d	n.d	n.d
*Salmonella* spp. (log CFU g^−1^) (10^−2^)	1.01 ± 0.01	n.d	n.d	n.d	n.d	n.d	n.d
Total yeast cells counts (log CFU g^−1^) (10^−4^)	2.10 ± 0.04^a^	1.73 ± 0.02^ab^	1.23 ± 0.22 ^b^	1.97 ± 0.08^ab^	1.59 ± 0.06^ab^	1.18 ± 0.18^b^	1.81 ± 0.10^ab^

^a^n.d **–** Not detected.

^b^Data represented as means ± SEM (n = 3). Mean values in the same row having different superscript letters (a, b c and d) are significantly different at *P* ≤ 0.05.

### 3.3. Gut microbiota

At the end of the experiment, the highest levels of bacterial total plate count (TPC) were recorded in fish fed on Diet 4 (1.94 × 10^−4^ log CFU g^−1^) and lowest in Diet 6 (Table 4). Levels of TPC were significantly higher in fish fed on Diet 4 followed by the control diet and Diet 1 (*P* < 0.05). TPC was significantly affected by the diet (*P* < 0.05). Yeast levels were significantly lower in the control group (*P* < 0.05). However, fish fed on yeast-based diets (Diet 1, 2 and 3) had a significant higher number of yeast cells counts (*P* < 0.05) compared to the control. *Bacillus* spp. counts in the gut were significantly higher in all the fish fed on probiotic treated diets compared to the control. Fish fed on Diet 2 had the highest levels of *Bacillus* spp. in the gut (*P* < 0.05).

**Table 4.** Gut microbiota of *O. niloticus* fed on *S. cerevisiae and B. subtilis* treated diets in low input ponds.

Parameter	Diet						
	Diet 0	Diet 1	Diet 2	Diet 3	Diet 4	Diet 5	Diet 6
Total plate count (log CFU g^−1^) (10^−4^)	1.85 ± 0.14^ab^	1.87 ± 0.02^ab^	1.70 ± 0.02^ab^	1.73 ± 0.02^ab^	1.94 ± 0.02^a^	1.75 ± 0.03^ab^	1.63 ± 0.03^b^
Yeast (log CFU g^−1^) (10^−4^)	1.35 ± 0.02^a^	1.63 ± 0.05^b^	1.62 ± 0.04^b^	1.64 ± 0.06^b^	1.34 ± 0.02^a^	1.36 ± 0.02^a^	1.35 ± 0.03^a^
*Bacillus* spp. (log CFU g^−1^ (10^−4^)	1.48 ± 0.03^a^	2.01 ± 0.06^b^	2.44 ± 0.17^b^	2.01 ± 0.07^b^	2.05 ± 0.12^b^	2.30 ± 0.05^b^	2.03 ± 0.10^b^

^a^Data represented as means ± SEM (n = 3). Mean values in the same row having different superscript letters (a, b and c) are significantly different at *P* ≤ 0.05.

## 4. Discussion

### 4.1. Hemato-immunological parameters

Hematological parameters are indicators for fish health, well being, physiological responses, nutritional status and environmental conditions [27,45,46]. In this study, blood samples from fish fed on probiotic treated diets contained a significantly higher number of hemoglobin, red blood cells (RBC) and white blood cells (WBC) compared to the control. Particularly, fish fed on Diet 2 and Diet 5 exhibited higher levels of hemoglobin compared to other treatments. Similarly, increase in levels of hemoglobin have been reported in Nile tilapia fed on *S. cerevisiae* treated diets and *Bacillus* spp. based probiotics [31,47–50].

Red and white blood cells are essential components in both innate and adaptive immune response and a higher abundance indicates a stronger immune system [51]. Levels of RBC and WBC were significantly higher in fish fed on Diet 2 and Diet 5. Likewise, higher levels of RBC and WBC were observed in a study on Nile tilapia fed on *S. cerevisiae* treated diets at a dosage between 1 to 6 g kg^−1^ indicating that increase in probiotic supplementation may reflect improved immunity of fish [28]. On the contrary, Ali et al. [52] found no remarkable differences in hematological parameters of Nile tilapia fed on commercial probiotic, Biogen (containing *B. subtilis*) and attributed it to the differences in the composition of Biogen and the dosage levels. Therefore, improvement in hematological parameters in the fish fed probiotic treated diets in the current study indicates the role of single species probiotics in stimulating immune responses of fish under stressful conditions, thereby reducing the deleterious effects caused by biological, chemical and physiological stress in the culture system [19,53,54].

In the current study, we observed an increase in serum protein and serum albumin with an increase in probiotic dosage. A similar trend was observed in *O. niloticus* fry fed on baker’s yeast probiotic up to 1 g kg^−1^ [27] and adult *O. niloticus* fed on baker’s yeast up to 6 g kg^−1^ [28]. According to Wiegertjes et al. [55], a high level of serum protein and serum albumin are associated with strong innate immune response in fish. Moreover, a study carried out on *Labeo rohita* fed on a mixture of probiotics (*B. subtilis, Lactococcus lactis* and *S. cerevisiae*), resulted in an increase in the level of serum protein, albumin and globulin with a reduction in A/G ratio [54]. This study realized a significant increase in globulin accompanied by a significant decrease in Albumin/Globulin (A/G) ratio in fish fed on Diet 2 with the control having the highest A/G ratio. This is an indication that probiotic administration promoted the immune system of Nile tilapia. Similarly, increase in globulin levels have been reported in Nile tilapia fed on *Bacillus spp*. based probiotics [49,56] and a reduction of A/G ratio have been demonstrated in rainbow trout (*Oncorhynchus mykiss*) fed on multi-strain probiotic bacteria (*Bacillus* sp., *Pediococcus* sp., *Enterococcus* sp., *Lactobacillus* sp.) [57]. Therefore, the increase in the total serum globulin and decrease in the A/G ratio realized in our study could be attributed to a high level of specific immunoglobulin (antibody) in the blood of the fish hence enhanced protective mechanisms for fish [29].

Serum lysozyme was higher in fish fed on probiotic treated diets than the control. This could be an indicator of the ability to kill pathogenic bacteria by breaking down the cell wall of both gram positive and gram negative bacteria [18,30,58,59]. *Saccharomyces* spp. have been found to trigger serum lysozyme level in *O. niloticus* and other teleosts [27,50]. Similarly, higher serum lysozyme activity has been reported in Nile tilapia [30,60–63], common carp (*Cyprinus carpio*) [64], rainbow trout (*O. mykiss*) [57,65] and brown trout (*Salmo trutta*) [66] fed on various probiotics. On the contrary, high doses of probiotics (*Saccharomyces cerevisiae* (6 g kg^−1^) and *Bacillus subtilis* (15 g kg^−1^) led to low lysozyme activity in the current study. A similar scenario was reported for rainbow trout fed on diets supplemented with a commercial probiotic (*B. subtilis* + *B. cereus toyoi*) at 0.03–0.06% of the diet [67]. Low lysozyme activity has been associated with immunosuppression after long-term exposure to immunostimulants [68]. This could be the case in the current study. However, the contradictory effect of probiotics on the immune response of fish could also be related to differences in microbial concentration, viability, type of probiotic used and duration of treatment [16,69].

### 4.2. Fish muscle microbiological content

Fish are always in contact with their living environment which has high concentrations of different kinds of pathogens (bacteria, viruses and parasites) and stressors (chemical and physical) [10,70]. Therefore, the epithelial and mucosal barrier of the skin, gills and alimentary tract are extremely important barriers against pathogens and diseases in fish [18,70,71]. Most organisms in fish culture system are either saprophytic or pathogenic and enter the fish through the body and intestine surfaces before causing infections and are often associated with the postharvest quality of fish [14,16,72].

Fish fed on probiotic treated diets had lower microbial load than the control in the current study. *E. coli* were significantly higher in the control compared to other treatments while faecal coliform and *Salmonella* spp. were only detected in the control. Nevertheless, total yeast cells counts were detected in all treatments but were significantly lower in fish fed on Diet 2 and 5 respectively. This could be attributed to the fact that more mucus could have been secreted in the fish fed probiotic treated diets compared to the control. Feeding fish on probiotics leads to sufficient mucus secretion by the fish inhibiting transfer of microorganisms from the environment to the flesh. The mucus layer in fish skin contains lectins, pentraxins, lysozymes, complement proteins, antibacterial peptides and immunoglobulin M (IgM) which have an important role in inhibiting the entry of pathogens to the fish [18,70].

### 4.3. Gut microbiota

In the this study, we realized an increase in yeast cells in the gut of fish fed on Diet 1, 2 and 3 and an increase in *Bacillus* spp. counts in the gut of the fish fed on Diet 4, 5 and 6. This indicates that the respective probiotic led to an increase of the respective bacteria in the gut of the host. Fish fed probiotic treated diets had less pathogenic bacterial load in their gut and is a sign of enhanced immunity. According to Ringø et al. [16], the increase of beneficial microbes in the gut is an indication of the positive role probiotics play in improving the intestinal microbial balance of the host by replacing harmful bacteria with beneficial bacteria. Gut microbiota often plays an important role in preventing pathogens from colonizing the gut, but this depends on the type of probiotic used [16]. Results of the current study confirm earlier studies that demonstrated the antagonistic effect of *Bacillus* spp. against pathogenic bacteria by competing for the same nutrients and adhesion sites resulting in stimulation of the immune system and improvement of the intestinal microbial balance [16,69,73,74].

Presence of yeast cells in the gut of experimental fish in the current study agrees with results of He et al. [68], who observed growth stimulation of a variety of beneficial bacteria and yeasts in the gut of hybrid tilapia (*Oreochromis niloticus* ♀ × *O. aureus* ♂) that were fed commercial *S. cerevisiae* product (DVAQUA®). Probiotic adhesion in the fish gut is very important in improving intestinal microbial balance and modulation of non-specific immunity [75]. The presence of the administered probiotics in the gut of the fish in the current study is an indicator that the probiotics remained viable in the gut of fish during the growth period and were able to survive the digestion process. This shows that the two probiotics were beneficial to the host [14,24]. The adhesion of the probiotic in the gut can be related to improved internal environmental conditions for beneficial microbial growth and a suitable environment that inhibits the growth of harmful microbial cells in the intestine of the host [76]. Navarrete and Tovar-Ramrez [21]; Ringø et al. [16] and Li et al. [14] established that probiotics have strong adhesion to fish intestinal mucus and outcompetes pathogenic microorganisms for available receptor sites for attachment in fish gut.

## 5. Conclusion

This study determined the optimum doses of baker’s yeast, *S. cerevisiae* and *B. subtilis* for incorporation into feed for Nile tilapia cultured in low input ponds that might be helpful in non- specific immune system modulation of the fish in challenging environments such as low-input pond systems. Continuous administration of dietary *S. cerevisiae* at 4 g kg^−1^ and *Bacillus subtilis* at 10 g kg^−1^ improved the innate immune condition, lowered fish muscle contamination with pathogenic bacteria and modulated the gut microbiota of *O. niloticus. S. cerevisiae* led to better immunity compared to *B. subtilis* demonstrating that it’s a more effective probiotic compared to bacteria. The microbial content of the fish in the current study was lower than the recommended microbiological limits for fish and can be regarded as safe for human consumption. Future studies should be focused on challenge trials using the recommended probiotic levels for Nile tilapia cultured in stressful conditions to establish their resistance to environmental stress and morphometric assessment of the intestinal villi to evaluate the effect of probiotics on gut morphology.
